# Triple threat: adiposity, aging, atrial fibrillation

**DOI:** 10.18632/aging.101318

**Published:** 2017-11-01

**Authors:** Justus M. Anumonwo, José Jalife, Daniel R. Goldstein

**Affiliations:** The Department of Internal Medicine/Cardiovascular Medicine, University of Michigan, Ann Arbor, MI 48109, USA

**Keywords:** aging, inflammation, adiposity

As the number of older people throughout the globe continues to grow, the health- related consequences of the aging population will dominate medical care. Chronic inflammation underpins several age-related diseases such as atherosclerosis, Alzheimer's Disease, sarcopenia, and arthritis. The origins of chronic inflammation with aging are not clear but increasing adiposity may be a contributor. More than a third of individuals aged greater than 60 are obese with a particular increase in intra-abdominal fat. Prior experi-mental studies in mice have revealed that aging induces a more inflammatory profile in the visceral fat consisting of inflammatory (termed M1) macrophages [1]. Furthermore, the age-enhanced production of inflammatory cytokines such as tumor necrosis factor (TNF)-ɑ, which induce insulin resistance during obesity [2], in visceral adipose tissue depends on the expression of Toll like receptor 4, a key innate immune receptor [1].

One potential highly morbid consequence of obesity is atrial fibrillation (AF). AF is the most common arrhythmia, affecting nearly five million patients across the US and the number one cause of hospitalizations from arrhythmias [3]. The incidence of AF is projected to double over the next two decades. Clinical studies have associated increased body mass index with AF. Specifically, with every unit increase in body mass index there is up to an 8% increase in the development of AF [4]. While obesity occurs with other risk factors for AF, including hypertension, atherosclerosis, diabetes and sleep apnea, an ovine AF model demonstrated that obesity alone can substantially enhance AF [5]. Given the epidemic of obesity across the globe and particularly within the US, we urgently need to understand the mechanisms by which obesity enhances AF.

Inflammation could be an underlying mechanism connecting adiposity to AF. Clinical studies have associated several circulating inflammatory mediators with AF, including C-reactive protein (CRP), interleukin (IL)-1β, IL-6, TNF-ɑ, and immune comp-lement activation with persistent AF [6]. Epicardial fat is a major source of adipokines, inflammatory cytokines and free fatty acids, which contributes to fibrotic remodeling within the atrial myocardium [7]. Macrophages and neutrophils, key cellular mediators of inflammation, may contribute to AF by infiltrating the atria or the epicardial fat, releasing reactive oxygen species, producing inflammatory cytokines, chemokines, metalloproteinases or myeloperoxidases. Inflammation is also critical for insulin resistance in experimental models of diet-induced obesity [3]. Increasing adiposity leads to the recruitment of macrophages into the fat depots. Macrophages, along with adipocytes, produce inflammatory mediators including TNF-ɑ, which may mediate insulin resistance. Thus, inflammation occurs in obesity and AF, yet the inflammatory pathway linking obesity to AF has not been identified.

Clearly, future investigations will be required to unravel how increased adiposity, either from diet or from aging, leads to AF. To comprehensively address this highly biomedically relevant issue will require a combination of experimental approaches. Specifically, murine models will allow one to genetically delete or over express a pathway of interest in a specific cell type. Large animal models, such as the sheep, in which AF can be sustained for weeks [8] will allow for the examination of novel therapeutics in dosages that may be effective in humans. Use of in vitro cultures with human cardiomyocytes derived from stem cells will permit one to examine whether inflammatory mediators produced by adipose tissue remodel human atrial ion channels to promote AF. Through such a combined strategy, one hopes to elucidate novel inflammatory pathways and novel anti-inflammatories to mitigate the AF promoting effects of adiposity that occur with aging and obesity.
